# Topographic Organization of Median Nerve Muscular Branches Reveals Frequent Shared Motor Pathways in the Human Forearm

**DOI:** 10.3390/brainsci16101068

**Published:** 2026-10-05

**Authors:** Alexandre Merí-Vived, Pedro Víctor López Plaza, Antoni Morral, Manuel Llusà-Pérez, Marcel García-Cuesta, Aroa Casado Rodríguez

**Affiliations:** 1 Facultat de Ciències de la Salut Blanquerna, Universitat Ramon Llull, 08025 Barcelona, Spain; pedrovictorlp@blanquerna.url.edu (P.V.L.P.); antonimf@blanquerna.url.edu (A.M.); 2 Unit of Anatomy and Human Embryology, Department of Surgery and Medical-Surgical Specialties, Faculty of Medicine and Health Sciences, University of Barcelona (Campus Clínic), 08036 Barcelona, Spain; mllusa@ub.edu (M.L.-P.); m.garciacuesta@ub.edu (M.G.-C.); 3 Faculty of Medicine, University of Vic—Central University of Catalonia, 08500 Vic, Spain; 4 Department of Medicine and Life Sciences, Pompeu Fabra University, 08003 Barcelona, Spain

**Keywords:** median nerve, motor branches, peripheral nerve, neuroanatomy, forearm, branching architecture, anatomical variation, nerve transfer, peripheral nerve surgery

## Abstract

**Highlights:**

**What are the main findings?**

- Seventy coordinate-defined muscular branches were described across 16 right forearms using proximodistal rank; the observed distribution is descriptive and does not establish independent reproducibility of branch location.
- Nearly half of the muscular branches (45.7%) supplied more than one target muscle, indicating that shared motor pathways are a common feature of median nerve branching.

**What are the implications of the main findings?**

- Branch-order maps describe where a muscular branch originates from the median nerve; they should not be interpreted as maps of downstream bifurcations, motor entry points, or intramuscular arborization.
- Branch-origin maps may inform anatomical exploration, but shared pathways and variable muscular destinations require case-specific identification. Improved surgical localization, safety, and patient outcomes were not evaluated.

**Abstract:**

**Background/Objectives**: Classical descriptions of median nerve motor anatomy often emphasize muscle-specific destinations, although a single muscular branch may divide to supply several muscles. This can obscure the hierarchical relationship between branch origin and downstream ramification. We therefore quantified the two-dimensional topography of muscular branches arising directly from the median nerve and analyzed each branch origin as a single spatial event linked to its subsequent muscular destinations. **Methods**: Sixteen fresh, non-frozen right upper limbs from 16 independent adult body donors (8 male and 8 female; mean age, 82.2 ± 9.6 years) underwent standardized dissection. Cartesian coordinates were assigned only to the origin of each first-order muscular branch from the median nerve. Branches were ordered proximodistally and linked to downstream subbranch count and target muscles. Coordinates were normalized to bistyloid distance (RPX) and forearm length (RPY). Donor-clustered bootstrap confidence intervals and mixed-effects models with donor-specific random intercepts summarized spatial variation across branch rank, with the longitudinal association interpreted descriptively because rank was defined proximodistally. **Results**: Seventy muscular branches generated 274 recorded downstream subbranches and 146 unique branch–muscle associations. Limbs contained a median of 4 muscular branches (range, 2–6), and 32/70 branches (45.7%) supplied two or more target muscles; this proportion includes the anterior interosseous nerve when its origin met the study’s branch-centered definition. The first branch participated in pronator teres innervation in all 16 limbs and was exclusive to that muscle in 11 (68.8%); all five sixth-ranked branches observed in this sample supplied flexor digitorum superficialis. As expected from the ranking definition, increasing branch order was descriptively associated with a more distal origin (β = 0.084; 95% CI, 0.063–0.104) and was associated with a higher normalized transverse coordinate (β = 0.077; 95% CI, 0.050–0.103; *p* < 0.001). Branch order explained substantially more longitudinal than transverse variance (marginal R^2^, 0.444 vs. 0.183). **Conclusions**: Proximodistally ranked median nerve branch origins were associated with frequent and variable downstream multi-muscle pathways. The rank–longitudinal coordinate association does not independently establish anatomical reproducibility. Treating the muscular-branch origin as the coordinate-level unit preserves the anatomical hierarchy, prevents pseudoreplication of shared coordinates, and provides an anatomical framework with potential relevance to peripheral nerve surgery; clinical benefits remain untested.

## 1. Introduction

The topographical organization of the median nerve in the forearm is clinically important because nerve repair, nerve transfer, selective neurotomy, and nerve-sparing dissection require identification of small motor pathways while preserving adjacent neural structures. The median nerve supplies most of the flexor-pronator musculature and contributes critically to pronation, wrist and finger flexion, and thumb function [1]. For selective procedures, however, knowledge of the final muscle destination is not sufficient: the surgeon must also understand where a muscular branch arises from the parent nerve and how that branch subsequently ramifies.

The muscular branches of the median nerve show substantial interindividual variability. Differences have been reported in branch number, level of origin, sequence of emergence, common trunks, and muscular distribution [2,3]. Canovas et al. quantified the levels of origin of median nerve muscular branches and reported marked variability for branches to pronator teres, palmaris longus, flexor carpi radialis, and flexor digitorum superficialis, whereas the anterior interosseous nerve and some of its branches were comparatively less variable [2]. Caetano et al. likewise described considerable heterogeneity and found that only a minority of dissected limbs reproduced the classical branching pattern presented in standard anatomical descriptions [3].

This variability becomes especially relevant when a motor pathway must be identified selectively. Parot and Leclercq documented frequent common trunks between branches supplying different muscles and emphasized the consequences for selective neurectomy [4]. Distally, forearm muscles may receive several primary motor branches and multiple terminal nerve entry points [5], and terminal nerve entry points show muscle-specific distributions [6]. These observations demonstrate that branch origin, extramuscular ramification, terminal nerve entry, and intramuscular arborization are related but anatomically distinct levels of organization.

Previous anatomical studies have localized neural structures using several reference strategies. Roberts et al. mapped optimal injection regions for forearm flexor muscles relative to reproducible surface and osseous landmarks [7]. Ye et al. and Park et al. characterized motor entry or intramuscular branch regions of the flexor digitorum superficialis [8,9], Hwang et al. described nerve entry points of the flexor digitorum profundus using normalized forearm coordinates [10], and Jeon et al. quantified the anterior interosseous nerve branch point and entry locations to deep flexor-pronator muscles [11]. These studies are clinically informative, but their primary anatomical unit is generally a distal neural destination or entry region rather than the first-order origin of a muscular branch from the median nerve trunk.

That distinction is methodologically important. When one muscular branch divides downstream to supply several muscles, assigning the same origin coordinate to each muscle-level record duplicates a single anatomical event and artificially increases the apparent number of spatial observations. Conversely, a single target muscle may receive input from several distinct muscular branches. A topographic analysis that seeks to describe branching architecture should therefore preserve the muscular branch origin as the coordinate-level unit while linking that origin to all of its downstream ramifications and muscular destinations.

Normalized Cartesian coordinates provide a practical framework for comparing these branch origins across specimens of different sizes [8,10,11]. Their value in this context is not only two-dimensional localization, but also the ability to analyze the spatial sequence of branch origins without conflating those origins with downstream bifurcations or muscle-entry points.

The present study used a branch-centered analytical framework to describe the distribution of first-order muscular-branch origins by proximodistal rank, characterize transverse variation, and quantify how frequently a single branch origin gave rise to shared multi-muscle pathways. Because rank was assigned according to proximodistal position, its association with longitudinal coordinates was treated as descriptive rather than as an independent test of anatomical order. The substantive anatomical questions concerned the locations and dispersion of branch origins and the relationship between these origins and their downstream muscular destinations. Each first-order branch origin was treated as one spatial observation to avoid duplicating coordinates across muscle-level records.

## 2. Materials and Methods

### 2.1. Study Design

A cross-sectional observational cadaveric study was conducted to characterize the origins and downstream ramifications of muscular branches arising directly from the median nerve in the anterior forearm.

Standardized dissection was organized around two linked anatomical levels. A muscular branch was operationally defined as a first-order motor branch arising directly from the median nerve trunk. Only the point of origin of this branch from the parent nerve was assigned Cartesian coordinates. Any subsequent division distal to that origin was classified as a downstream subbranch or ramification and was not assigned an independent origin coordinate.

Branch order denotes the sequential proximodistal position of each first-order muscular branch arising from the median nerve. It does not imply homology of muscular destination between donors; for example, a second-ranked branch in one donor was defined by its position in the sequence rather than by a requirement to supply the same muscle as the second-ranked branch in another donor. The muscular branch origin was therefore the coordinate-level analytical unit, whereas muscular destination was analyzed through unique branch–muscle associations. This hierarchical definition prevented duplication of shared origin coordinates when a branch divided toward multiple muscles. Here, first-order describes a direct departure from the median nerve trunk, whereas branch order (1–6) describes the proximodistal rank among those direct departures. A branch ranked second or sixth is therefore still a first-order branch in the anatomical hierarchy. Since rank is assigned by longitudinal position, a positive rank–RPY association is partly inherent in the definition and does not independently validate reproducibility.

The study was approved by the Research Ethics Committee of the Blanquerna Faculty of Health Sciences, Universitat Ramon Llull (CER-FCSB; Approval No. 2025-09-03). All anatomical specimens were obtained through the Body Donation Programme of the Faculty of Medicine and Health Sciences, University of Barcelona (Campus Clínic, Barcelona, Spain), and were used in accordance with the ethical principles and institutional regulations governing body donation for teaching and scientific research.

### 2.2. Anatomical Specimens

Sixteen fresh, non-frozen right upper limbs from 16 independent adult body donors were included. Using one right limb per donor reduced laterality-related variability, ensured one biological replicate per donor, and avoided within-donor pseudoreplication. The sample comprised 8 male and 8 female donors; mean age was 82.2 ± 9.6 years, median age was 84.5 years (interquartile range, 79.8–88.3), and the range was 63–98 years. Specimens were eligible when the anterior compartment was intact and permitted complete identification of the median nerve and its branches. Prior surgery, traumatic injury, deformity, gross anatomical abnormality, or tissue deterioration interfering with dissection constituted exclusion criteria.

In each specimen, all muscular branches arising directly from the median nerve and ultimately supplying the following structures were identified: pronator teres, flexor carpi radialis, palmaris longus, fasciculus accessorius musculi flexoris pollicis longi (Gantzer’s fascicle), flexor pollicis longus, pronator quadratus, flexor digitorum profundus, and flexor digitorum superficialis.

Across the 16 limbs, 70 coordinate-defined muscular branches were identified. These branches generated 274 recorded downstream subbranches and were represented by 186 linked muscle-level records in the source database. After repeated labels for the same target muscle within a given muscular branch were collapsed, 146 unique branch–muscle associations remained. These quantities represent different anatomical levels and were not treated as interchangeable observations.

### 2.3. Anatomical Dissection

All specimens were dissected according to a standardized anatomical protocol. Before dissection, the upper limb was positioned with the elbow fully extended and the forearm in full supination to maintain a consistent anatomical position throughout the procedure.

A longitudinal skin incision was made along the anterior aspect of the forearm, followed by layer-by-layer dissection to expose the antebrachial fascia. After opening the fascia, the median nerve was identified and dissected from its entry into the forearm to its distal portion while preserving its relationships with adjacent muscular and neurovascular structures.

After exposure, the median nerve was traced to identify every muscular branch arising directly from the main trunk. Each muscular branch was assigned a proximodistal order and followed through all visible downstream bifurcations to its target muscle or muscles. For downstream counting, each anatomically distinct daughter branch arising distal to the first-order origin was counted separately, including terminal twigs when they remained macroscopically distinguishable before entering a target muscle. Counting stopped at the muscle-entry point; intramuscular arborization beyond that point was not counted. The count therefore represents visible extramuscular ramifications rather than microscopic intramuscular branching. No optical magnification was used during dissection or subbranch counting. A branch retained one origin coordinate regardless of the number of downstream subbranches or muscular destinations. Muscular branches ultimately supplying pronator teres, flexor carpi radialis, palmaris longus, Gantzer’s fascicle, flexor pollicis longus, pronator quadratus, flexor digitorum profundus, and flexor digitorum superficialis were documented. A representative dissection showing the median nerve muscular branches and their downstream ramifications is provided in Video S1.

The anterior interosseous nerve (AIN) was recorded using the same branch-centered procedure as the other muscular branches. Its point of departure from the median nerve was documented as a branch origin, and the AIN was then followed distally to record its subsequent divisions and the muscle supplied by each division. Thus, the AIN was not treated as a separate analytical category: its origin, downstream branches, and muscular destinations were documented according to the same anatomical hierarchy applied throughout the study. Subsequent divisions toward flexor pollicis longus, pronator quadratus, or flexor digitorum profundus were considered downstream ramifications of that recorded origin rather than additional departures from the median nerve.

### 2.4. Anatomical Positioning and Coordinate Acquisition System

**Figure 1:**
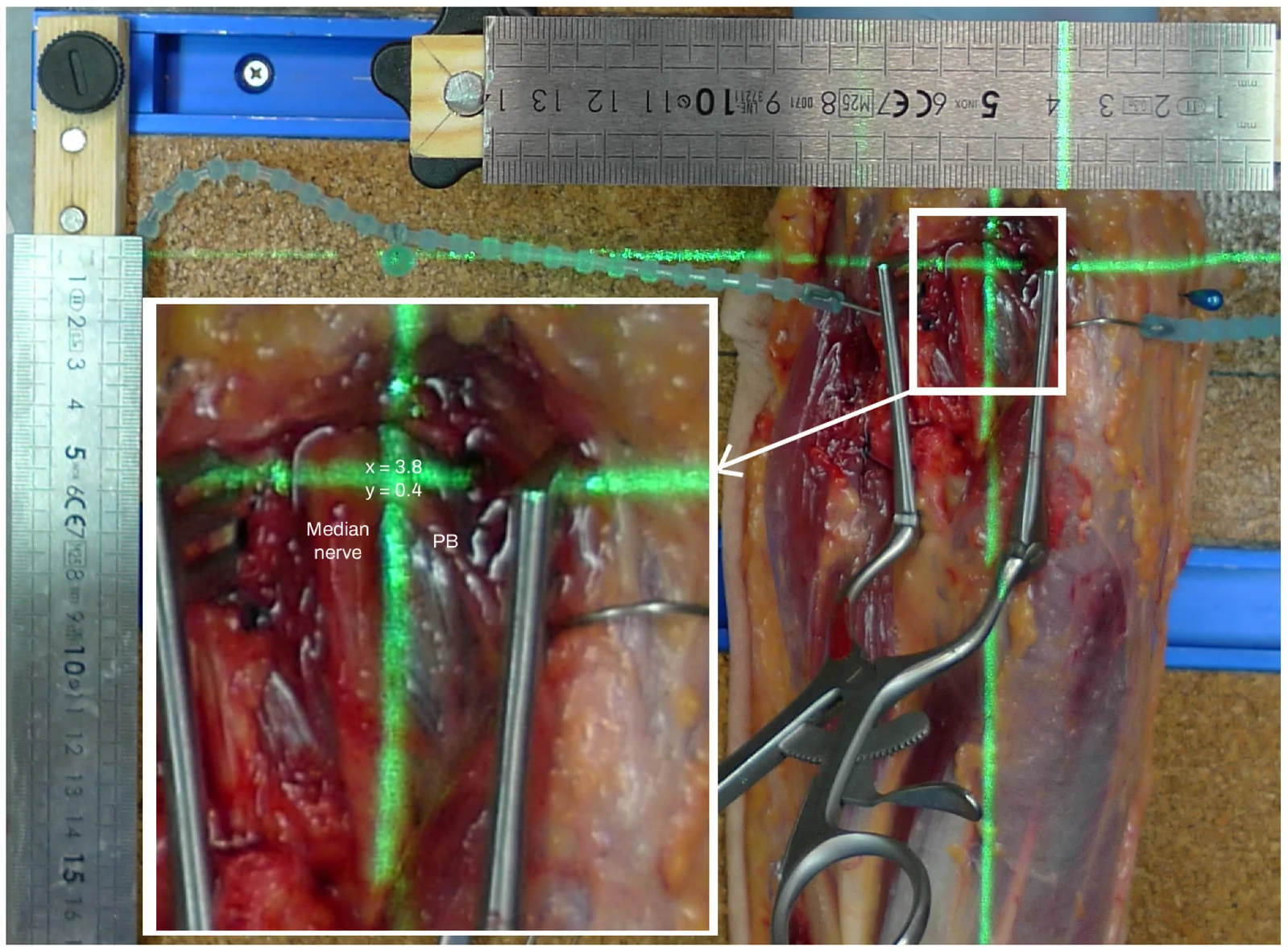
Experimental setup for standardized anatomical positioning, dissection, and spatial measurement. The enlarged inset shows the median nerve and a representative first-order branch origin projected onto the horizontal (X) and longitudinal (Y) laser reference lines; the rulers provide the corresponding metric coordinates.

Before and throughout dissection, each specimen was maintained in a constant anatomical position by securing it to a custom-designed platform developed to ensure complete immobilization during data acquisition (Figure 1). The upper limb was stabilized using two fixation points located proximally above the biepicondylar line and distally below the bistyloid line, thereby avoiding movement that could modify the spatial arrangement of the muscular branches.

**Figure 2:**
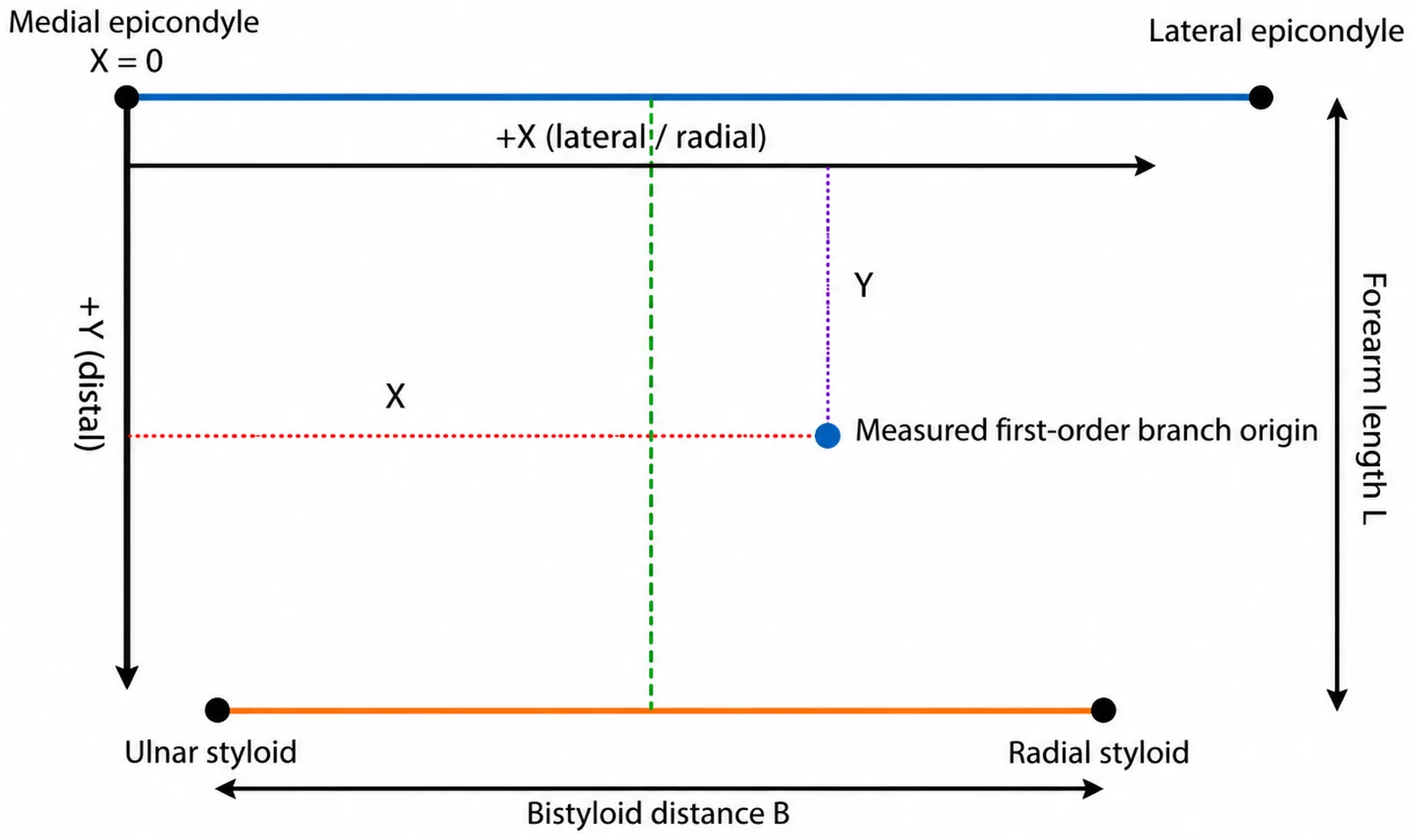
Schematic representation of the coordinate acquisition system. X = 0 is aligned with the medial epicondyle and increases laterally/radially, whereas Y = 0 corresponds to the biepicondylar line and increases distally. The blue line represents the biepicondylar reference line; the orange line, the bistyloid reference line; the green dashed line, the midpoint of the bistyloid distance; the red dotted line, the horizontal reference used to determine X; and the purple dotted line, the vertical reference used to determine Y. The blue dot indicates the measured first-order branch origin. Relative coordinates were calculated as RPX = X/B and RPY = Y/L, where B is the bistyloid distance and L is the forearm length. Schematic not to scale.

Measurements were obtained using a standardized Cartesian coordinate system (Figure 2). The forearm was immobilized between perpendicular horizontal and vertical rulers, and laser reference lines projected the location of each first-order muscular-branch origin onto both scales. The transverse coordinate was defined as X, with X = 0 aligned with the medial epicondyle and positive values increasing laterally (radially). The longitudinal coordinate was defined as Y, with Y = 0 at the biepicondylar line and positive values increasing distally toward the wrist. For each branch, the point measured was the direct origin of the branch from the median nerve; downstream bifurcations and muscle-entry points were not assigned independent coordinates.

**Figure 3:**
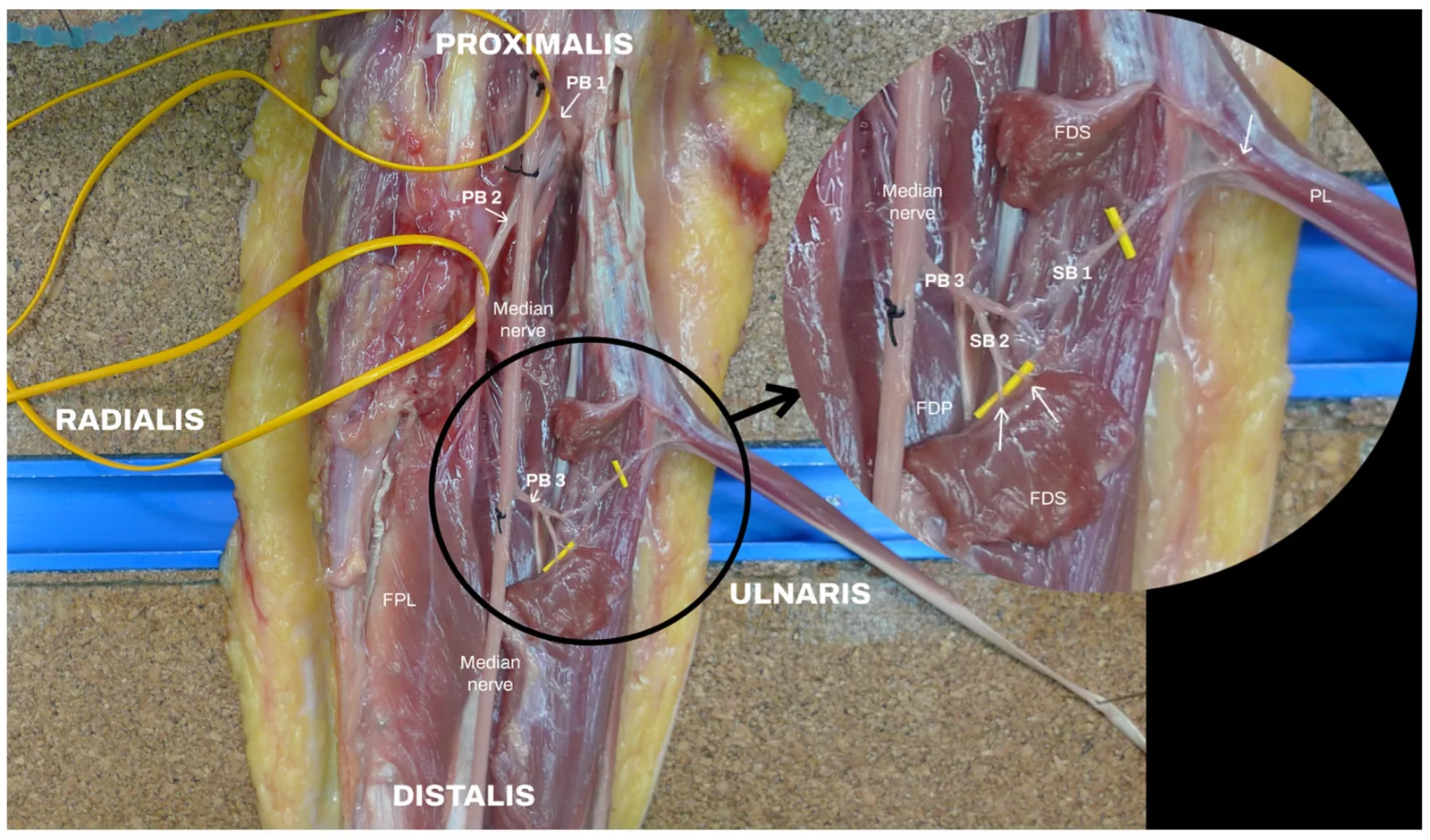
Representative forearm dissection illustrating the median nerve and a shared muscular branching pathway. FDS, flexor digitorum superficialis; FDP, flexor digitorum profundus; FPL, flexor pollicis longus; PL, palmaris longus; PB1-PB3, first-order principal branches arising from the median nerve; SB1-SB2, downstream subbranches. Anatomical directions (proximalis, distalis, radialis, and ulnaris) orient the specimen. The enlarged inset demonstrates the continuation of PB3 into downstream divisions and muscular destinations. White arrows indicate distal muscle-entry points; these are anatomically distinct from the first-order origins used for coordinate analysis.

Anthropometric measurements required for normalization included forearm length, biepicondylar distance, forearm circumference, bistyloid distance, and carrying angle. Forearm length was measured longitudinally from the biepicondylar line to the bistyloid line (Figure 3). The transverse coordinate was normalized to bistyloid distance and the longitudinal coordinate to forearm length, allowing comparison among specimens of different size. Bistyloid distance was selected as the transverse scaling variable because it provides a standardized distal forearm-width measure at the same distal reference level used to delimit forearm length and is less influenced by the breadth of the elbow region than biepicondylar distance. This normalization was intended as a size-adjustment procedure, not to imply that the medial-epicondylar X origin and the distal scaling landmark are anatomically coincident.

For normalization, RPX = X/B and RPY = Y/L, where X is the transverse distance from the medial-epicondylar zero, Y is the longitudinal distance from the biepicondylar line, B is the bistyloid distance, and L is forearm length measured between the biepicondylar and bistyloid lines. Because the X origin is defined proximally at the medial epicondyle, whereas B is the distal wrist width, RPX can exceed 1 when the transverse branch coordinate is greater than the bistyloid distance. Negative RPY values can occur when a measured branch origin lies slightly proximal to the biepicondylar reference line.

### 2.5. Anatomical Measurements and Normalization

For each first-order muscular branch, the origin, proximodistal order, total number of downstream subbranches, target muscle or muscles, and available entry characteristics were recorded. Each first-order branch origin yielded one transverse (X-axis) and one longitudinal (Y-axis) coordinate. Accordingly, spatial measurements refer exclusively to the origin of first-order muscular branches arising directly from the median nerve. Downstream ramifications contributed to the subbranch count and muscular-destination analysis but not to the coordinate dataset. Repeated database rows with the same donor, branch order, and origin coordinates were recognized as downstream records belonging to the same muscular branch.

As additional anatomical landmarks, the position of the median nerve at its intersections with the biepicondylar and bistyloid lines was recorded. The positions of the tendons of pronator teres, flexor carpi radialis, palmaris longus, flexor digitorum superficialis, flexor digitorum profundus, and flexor pollicis longus were also documented at the bistyloid line.

Muscular destination was summarized as a unique association between a muscular branch and a target muscle. Thus, several downstream subbranches from one muscular branch to the same muscle contributed one association, whereas a shared muscular branch supplying several muscles contributed one association for each distinct target. Longitudinal measurements were normalized to forearm length and transverse measurements to bistyloid distance; the resulting RPY and RPX variables were used in all spatial analyses.

### 2.6. Measurement Quality Control

Before data collection, the investigators completed a methodological calibration session to standardize identification of muscular branches, determination of their origin from the median nerve, and all anatomical measurements.

Linear measurements were obtained using a precision digital caliper (Absolute Digimatic 500-196-30, Mitutoyo Corporation, Kawasaki, Japan), with a resolution of 0.01 mm and an accuracy of ±0.02 mm. Spatial coordinates were acquired using the anatomical positioning and laser projection system described above, with the biepicondylar and bistyloid lines serving as fixed anatomical reference landmarks. All coordinate measurements were recorded to the nearest 0.1 mm.

All measurements were performed independently by two investigators (A.C.R. and A.M.V.) using the same protocol and anatomical landmarks. Each investigator performed three repeated observations. During acquisition, the two investigators compared their readings as a quality-control check; the maximum observed discrepancy between investigators was 0.2 mm, and in most measurements the readings were identical. Only the final averaged value was retained in the study database; the six individual readings were not archived. Consequently, a retrospective ICC or formal repeatability coefficient cannot be calculated from the available dataset, and the observed 0.2-mm maximum discrepancy should be interpreted only as an acquisition-stage quality-control observation, not as a formal estimate of reliability.

### 2.7. Statistical Analysis

Analyses were performed in R version 4.6.0 (24 April 2026 ucrt; R Foundation for Statistical Computing, Vienna, Austria), using the tidyverse suite for data management and visualization, lme4 for linear mixed-effects modeling, and performance for model-derived statistics. Reported *p* values are two-sided. Data-reduction rules were specified from the anatomical hierarchy before modeling. Branch order was modeled as an ordinal continuous predictor to summarize the average coordinate change across successive ranks, not muscle-specific homology between ranks. In particular, the RPY slope was interpreted descriptively because the predictor was constructed from proximodistal position; it does not test an independent hypothesis of longitudinal organization.

Muscular branches were identified by the unique combination of donor, branch order, and Cartesian origin. RPX was calculated by dividing the transverse coordinate by bistyloid distance, and RPY by dividing the longitudinal coordinate by forearm length. Branch count per limb, total downstream subbranches, number of distinct muscular targets, and unique branch–muscle associations were summarized descriptively.

For each branch order, mean RPX and RPY and their 95% confidence intervals were estimated using 10,000 donor-clustered bootstrap resamples. In each resample, donors—not individual branches or muscle-level records—were sampled with replacement, preserving all muscular branches contributed by each selected donor and thereby maintaining the within-donor dependence structure.

Linear mixed-effects models were fitted separately for the two normalized coordinates using the formulas RPX ~ branch_order + (1|donor) and RPY ~ branch_order + (1|donor), with branch order entered as a fixed continuous effect and donor as a random intercept. Models were estimated by restricted maximum likelihood (REML). Fixed-effect 95% confidence intervals were calculated using Wald intervals, and two-sided *p* values were obtained from the corresponding Wald test of the fixed-effect coefficient. Marginal and conditional R^2^ were calculated according to the Nakagawa framework using the performance package (marginal R^2^: variance explained by the fixed effect; conditional R^2^: variance explained by fixed and random effects). Results are reported as the change in normalized coordinate per one-order increment, together with 95% confidence intervals. The intraclass correlation coefficient (ICC) quantifies residual donor-level clustering in the spatial model. The difference between longitudinal and transverse marginal R^2^ values is descriptive and is influenced by the use of longitudinal position to define branch rank; it is not a formal comparison of reproducibility or localization accuracy.

Muscular destinations were analyzed descriptively because a muscular branch could supply several muscles and therefore could not be assigned to a single mutually exclusive muscle group. For each branch order, the proportion of muscular branches associated with each target muscle was calculated. Percentages may sum to more than 100% because shared muscular branches may reach several muscles.

The source data were also checked for internal consistency among repeated origin coordinates, branch order, and branch-level subbranch totals. Branch-level totals were used for complexity summaries, thereby avoiding multiplication of values repeated across downstream records.

### 2.8. Use of Generative Artificial Intelligence in Manuscript Preparation

During revision of the manuscript, ChatGPT (OpenAI, San Francisco, CA, USA; GPT-5.6 Sol) was used solely for linguistic editing and organizational improvement of the manuscript, including refinement of language, clarity, scientific writing, and overall text structure. The tool was not used for data analysis, generation or modification of study data or results, interpretation of findings, preparation of figures, or formulation of scientific conclusions. All AI-assisted edits were critically reviewed and verified by the authors, who retain full responsibility for the content of the manuscript.

## 3. Results

### 3.1. Donor Characteristics and Branching Architecture

The 16 fresh, non-frozen right forearms came from 16 independent donors (8 male and 8 female). Mean age was 82.2 ± 9.6 years, median age was 84.5 years (interquartile range, 79.8–88.3), and the range was 63–98 years. Each donor therefore contributed one biological replicate.

Seventy coordinate-defined muscular branches were identified. Limbs contained a mean of 4.4 ± 1.3 muscular branches and a median of 4 (interquartile range, 3.8–6.0; range, 2–6). These branches generated 274 recorded downstream subbranches, with a median of 2 per muscular branch (interquartile range, 1–5.8; range, 1–17). The source database contained 186 linked muscle-level records and 146 unique branch–muscle associations after repeated destinations within the same muscular branch were collapsed.

Thirty-eight muscular branches (54.3%) supplied one target muscle, whereas 32 (45.7%) supplied two or more muscles. Two shared branches reached as many as seven distinct muscular targets. Thus, shared multi-muscle pathways were not exceptional variants but a frequent component of the observed branching architecture. This frequency also demonstrates why downstream muscle-level records cannot be treated as independent coordinate observations.

### 3.2. Coordinate-Defined Sequence of Muscular-Branch Origins

**Figure 4:**
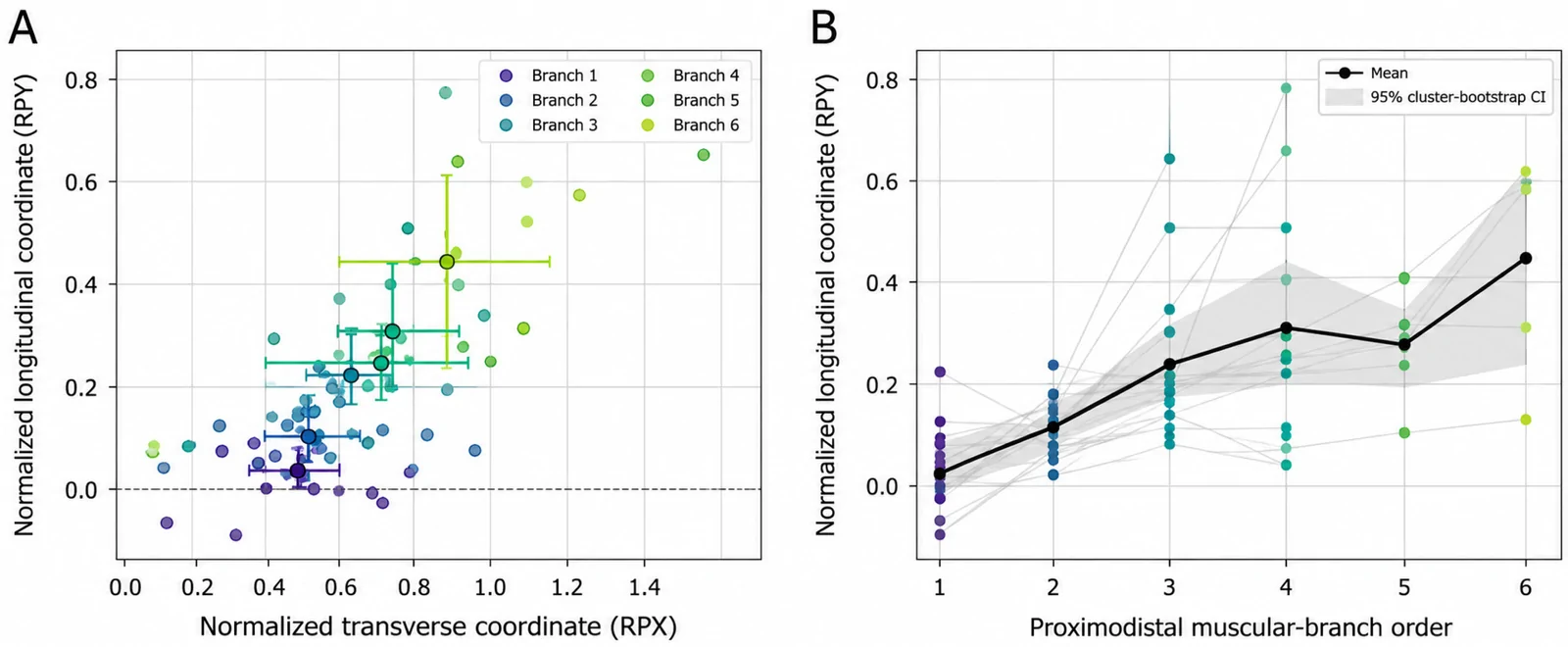
Normalized topography of the 70 median nerve muscular branches. (**A**) Individual origin coordinates and order-specific means with 95% donor-clustered bootstrap confidence intervals for RPX and RPY. (**B**) Within-donor sequence of RPY by proximodistal muscular-branch order; light grey lines connect branches from the same donor and the black line shows the order-specific mean. Later orders were absent in some donors, so denominators decrease from 16 at orders 1–2 to 5 at order 6. Within-donor longitudinal ordering follows from the ranking definition. Pooled means may be non-monotonic because the set of contributing donors changes across ranks; confidence intervals describe uncertainty in the means, not individual prediction regions.

Branch origins were ranked proximodistally within each donor (Figure 4; Table 1). Mean RPY was 0.032 for rank 1 and 0.440 for rank 6. The pooled means did not increase at every successive rank: mean RPY was 0.296 at rank 4 and 0.253 at rank 5, which were represented by different donor subsets. RPX also tended to increase with rank, although transverse dispersion widened among later and less frequently observed branches. These summaries describe the observed sample and do not establish a universal branch sequence.

In the mixed-effects analysis, each one-order increment corresponded descriptively to an RPY increase of 0.084 (95% CI, 0.063–0.104) and was associated with an RPX increase of 0.077 (95% CI, 0.050–0.103; *p* < 0.001). Branch order explained 44.4% of RPY variance but only 18.3% of RPX variance. Donor-level clustering was stronger transversely (ICC = 0.576) than longitudinally (ICC = 0.255). The longitudinal association is partly imposed by the definition of rank. The slopes and R^2^ values therefore describe the ranked coordinates but do not demonstrate independent reproducibility or accurate prediction of an individual branch location (Table 2). These ICC values quantify donor clustering, not agreement between investigators.

### 3.3. Muscular Ramifications by Branch Order

**Figure 5:**
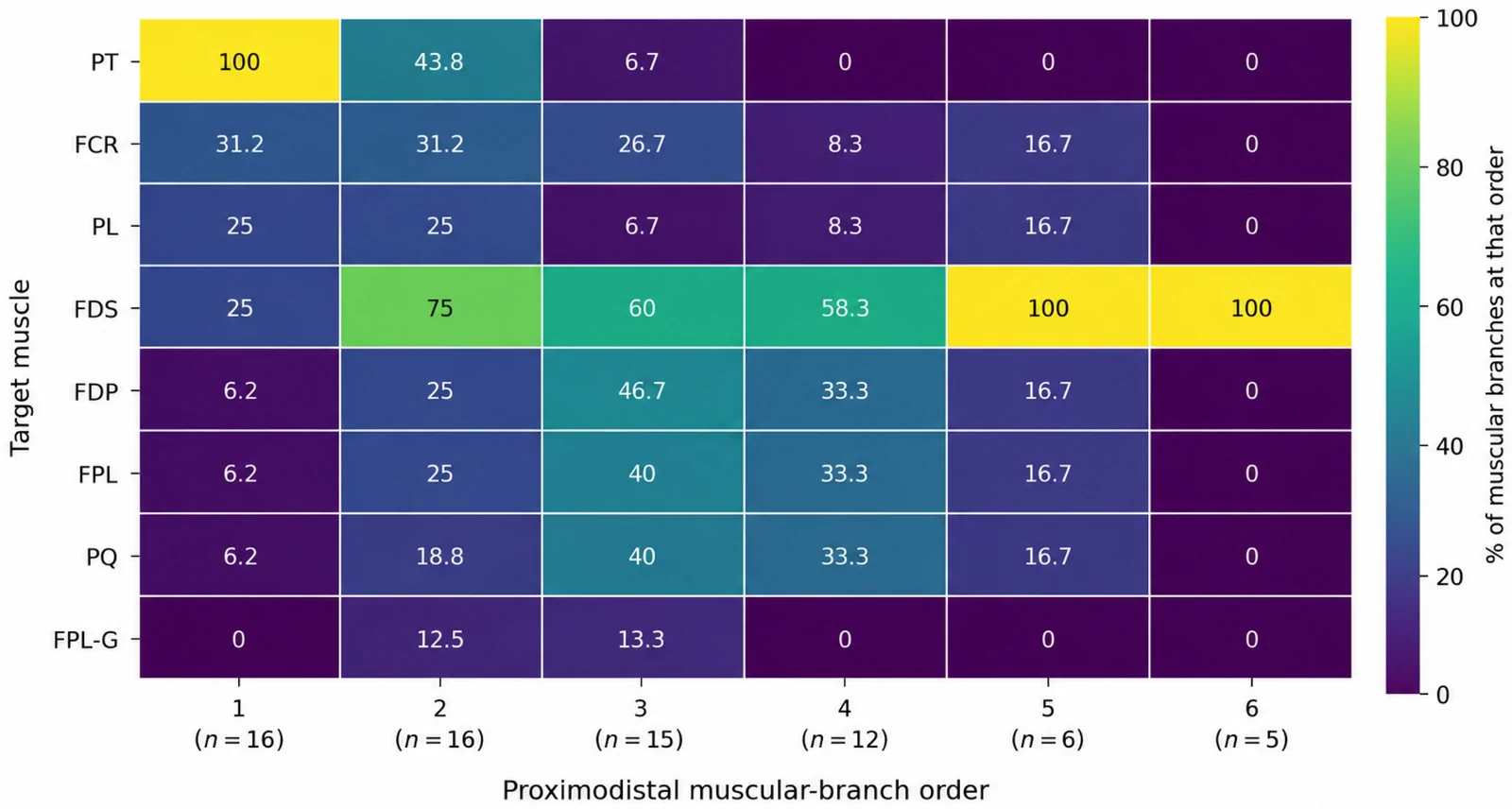
Target-muscle participation by proximodistal muscular-branch order. Values are percentages of muscular branches at each order associated with each muscle. Denominators were 16, 16, 15, 12, 6, and 5 branches for orders 1–6, respectively. Percentages within an order can exceed 100% in total because one muscular branch may supply several muscles.

The observed distribution of muscular destinations differed across branch ranks (Figure 5; Table 3), but no order was interpreted as a muscle-specific homologous branch across donors. Every first muscular branch participated in pronator teres innervation (16/16); in 11 limbs (68.8%) it supplied pronator teres exclusively, whereas in 5 it formed a shared pathway. Flexor digitorum superficialis participation increased from 25.0% at the first branch to 75.0% at the second and was present in every fifth- and sixth-order branch observed in this sample. All five observed sixth-order branches were exclusively associated with flexor digitorum superficialis.

Middle orders showed the greatest destination diversity. The third muscular branch was associated with flexor digitorum superficialis in 60.0% of limbs contributing that order, flexor digitorum profundus in 46.7%, flexor pollicis longus and pronator quadratus in 40.0% each, and flexor carpi radialis in 26.7%. The Gantzer fascicle was uncommon and appeared only among second- and third-order branches.

## 4. Discussion

This study describes the topography and downstream destinations of 70 first-order muscular branches in 16 right forearms. Shared pathways were frequent: 32 branches (45.7%) supplied more than one muscle. The coordinate distributions provide a descriptive reference for the sampled donors, but the association between proximodistal rank and longitudinal position is partly inherent in the ranking procedure. The principal anatomical contribution is the linkage of each direct branch origin to its downstream destinations while preserving the distinction between a branch origin and its later divisions.

**Figure 6:**
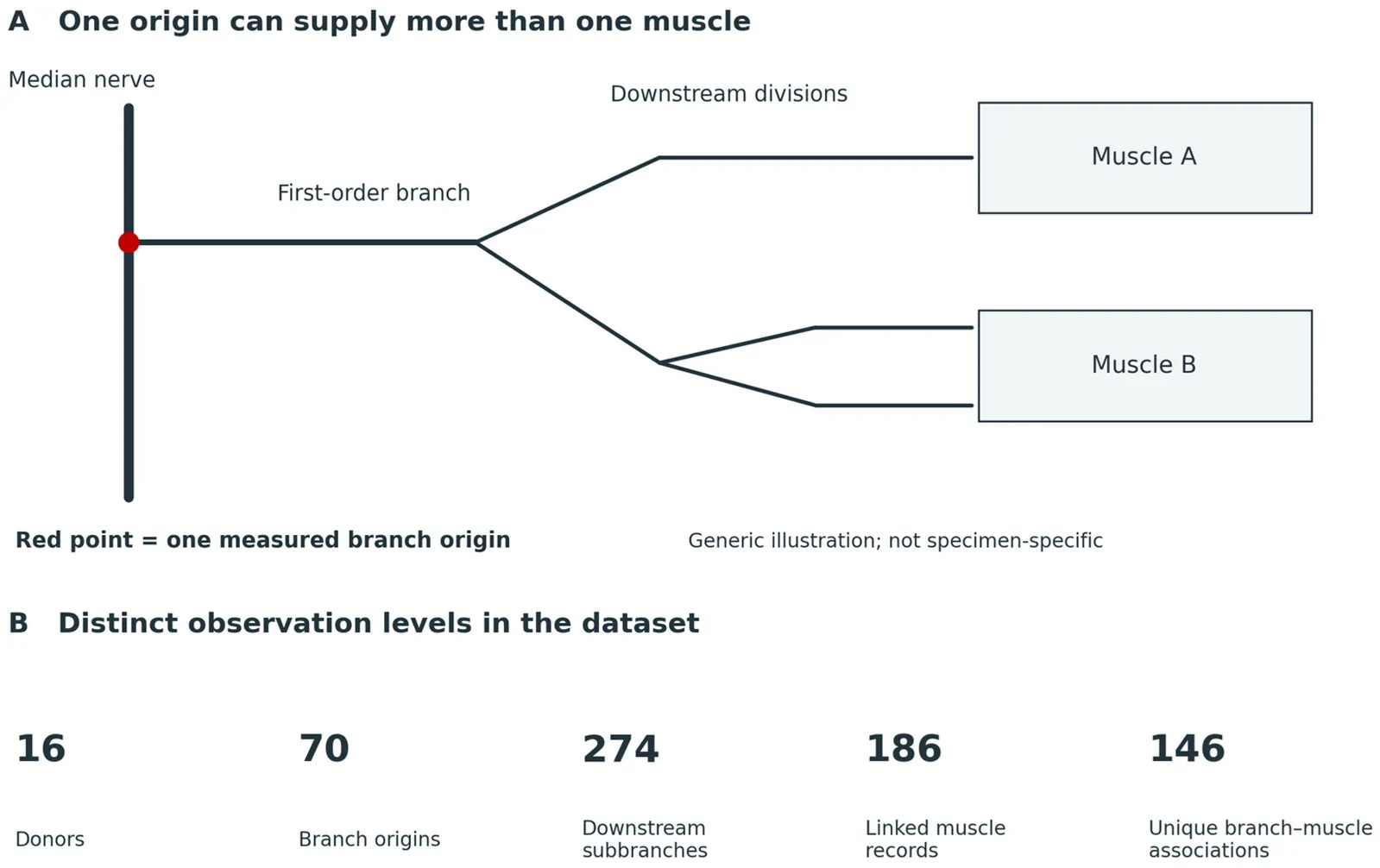
Conceptual representation of a shared motor pathway and the analytical hierarchy. (**A**) A single first-order branch origin from the median nerve leads through downstream divisions to more than one muscle. Generic targets A and B are illustrative, not a reconstruction of a particular specimen or an assertion about a specific muscle combination. Only the direct departure from the median nerve contributes an origin coordinate. (**B**) The study contains 16 donors, 70 branch origins, 274 downstream subbranches, 186 linked muscle-level records and 146 unique branch–muscle associations. These are distinct levels of observation, not interchangeable sample sizes. The revised schematic is not to scale.

The principal methodological contribution is the explicit separation of anatomical levels. A branch origin from the median nerve, an extramuscular bifurcation, a terminal nerve entry point, and an intramuscular arborization answer different anatomical and clinical questions. Previous studies have provided valuable maps for motor-point localization, injection planning, or distal neural anatomy [7,8,9,10,11], but those locations cannot be substituted for the branch-origin coordinates examined here. Conversely, the present coordinates should not be interpreted as direct injection maps. Preserving this hierarchy also prevents a shared origin from being counted repeatedly when one branch reaches several muscular targets (Figure 6).

The earliest part of the sequence was especially stable. The first muscular branch participated in pronator teres innervation in all 16 limbs, consistent with previous descriptions of pronator teres as an early motor destination of the median nerve [2,3]. Nevertheless, five of these first-ranked branches also supplied another muscle. Thus, ranking branches proximodistally does not imply an invariant relationship between branch order and muscular destination. This observation accords with the common trunks described by Parot and Leclercq [4] and with the broader variability of median-nerve branching reported in previous cadaveric series [2,3].

The 45.7% prevalence of multi-muscle branches is therefore more than a descriptive detail: it challenges any simplified interpretation of median-nerve motor anatomy as a set of independent muscle-specific branches. Shared pathways were common enough to be considered a characteristic of the observed architecture. Middle branch orders showed the greatest muscular diversity, with third-order branches frequently contributing to flexor digitorum superficialis, flexor digitorum profundus, flexor pollicis longus, and pronator quadratus. The same target muscle could also receive input from different first-order branches. Accordingly, a mean branch-origin coordinate cannot identify muscular destination deterministically.

Branch rank explained 44.4% of RPY variance and 18.3% of RPX variance in the fitted models. This contrast should not be interpreted as evidence that longitudinal anatomy is more reproducible: longitudinal position was used to assign rank, whereas transverse position was not. The transverse association is not imposed by that definition, but it also does not establish predictive accuracy. Order-specific means and confidence intervals describe the sample and uncertainty in its mean coordinates; they do not provide patient-specific localization bounds. Reproducibility of measurement and external anatomical consistency require separate assessments.

At the distal end of the observed sequence, all sixth-order branches in this sample supplied flexor digitorum superficialis. However, sixth-order branches occurred in only five donors, and fifth-order branches in only six. These later orders therefore have wider uncertainty and should not be interpreted as universal anatomical rules. The progressive reduction in denominator is visible in Figure 3 and should be considered when using order-specific coordinates for anatomical orientation.

The potential surgical relevance lies in anticipating shared destinations when a motor branch is exposed, rather than assigning a muscle identity from its rank or mean coordinate. During proximal forearm exposure or neurolysis, the observation that the first-ranked branch reached pronator teres in all 16 limbs may provide an anatomical expectation, but its additional destinations in five limbs argue for tracing the branch beyond its origin. This observation does not define a safe dissection corridor or a fixed distance from a surface landmark [2,3,4].

For selective neurectomy or nerve transfer, a shared proximal stem cannot be assumed to be specific to the intended muscle. Injury or division proximal to its downstream separation could affect more than one motor destination; anatomical continuity supports this possibility, but the present data cannot quantify the resulting weakness or functional loss. Distal tracing and functional assessment may help establish whether a muscle-specific component can be identified, but this study did not test the feasibility or safety of separating those components. The finding of multiple inputs to a muscle also does not establish that any one input is functionally dispensable [1,4].

Previous anatomical work has investigated the feasibility of transferring a flexor digitorum superficialis branch to recipient branches for wrist extension or pronation [1]. Our findings add a reason to verify all downstream targets before considering a branch for such a procedure. We did not measure branch length, calibre, axonal content, or tension-free reach, and therefore cannot identify a suitable donor branch or recommend a transfer on the basis of rank alone.

In traumatic nerve injury, repair or reconstruction, documenting the distal targets of a damaged shared stem may help explain involvement of several muscles and frame the anatomical assessment of the injury. Conversely, a branch-origin map does not describe intraneural fascicular topography and cannot guide selective fascicular dissection within the median nerve. These are potential applications of the anatomical findings, not operative techniques validated by this study.

The clinically relevant hypothesis is that recognizing shared pathways could reduce unintended compromise of uninvolved motor targets. No living patients, operative comparisons, localization-accuracy measurements, or postoperative functional outcomes were studied. Whether use of this information improves safety or patient outcomes requires prospective evaluation, including target-identification accuracy, unintended motor deficits and procedure-specific functional recovery.

Several aspects of the design strengthen the anatomical inference. Fresh, non-frozen specimens were examined in a standardized position; only one right limb from each donor was included, ensuring one biological replicate per individual; and each muscular branch was followed from its origin through visible downstream destinations. Coordinate-level and destination-level datasets were kept separate, donor-clustered bootstrap resampling preserved within-donor branch structure, and mixed-effects models explicitly accounted for donor-level clustering. Normalization to forearm dimensions further enabled comparison of branch-origin positions across specimens of different sizes.

The study also has limitations. The sample comprised 16 predominantly elderly donors, so uncommon variants and age-related differences may not be fully represented. Only right limbs were examined, which reduced laterality-related heterogeneity but precluded assessment of bilateral symmetry. The coordinate system was two-dimensional and did not capture depth, branch calibre, fascicular composition, axonal load, branching angle, or surgical reach. Later branch orders were represented by relatively few donors, limiting precision. Branch rank was modeled as an ordinal continuous predictor; the longitudinal association is partly inherent in the ranking definition, and the model does not characterize all nonlinear rank-specific effects. Muscular destinations were inherently multi-label and were therefore summarized descriptively rather than forced into mutually exclusive groups. The six individual repeated readings were not archived; although acquisition-stage comparison showed a maximum observed discrepancy of 0.2 mm between investigators and most readings were identical, formal interobserver or intraobserver reliability coefficients cannot be calculated retrospectively. The donor-clustering ICCs are not measurement-reliability estimates. The cadaveric design does not establish localization accuracy, operative safety, or improved patient outcomes. Finally, the study mapped branch origins and downstream destinations rather than complete intramuscular arborization or the full distribution of motor entry points; the findings should therefore not be extrapolated to injection targets. Observations involving Gantzer’s muscle should also be regarded as exploratory given the known variability of this accessory muscle [12].

Future studies should test the observed branch-order organization in larger, age-diverse, bilateral, and preferably multicentre anatomical samples. Three-dimensional reconstruction could determine whether depth and branching angle add clinically useful information, while measurements of calibre and fascicular composition could help distinguish branches that are anatomically close but functionally different. Direct coregistration with high-resolution ultrasonography or magnetic resonance neurography would be required to establish whether branch-order information improves in vivo localization beyond conventional anatomical landmarks. Independent validation is particularly important before any coordinate-based framework is used to guide selective procedures.

## 5. Conclusions

In 16 fresh, non-frozen right forearms, 70 first-order median nerve muscular branches generated 274 downstream subbranches, and 45.7% supplied more than one target muscle. The first-ranked branch participated in pronator teres innervation in all 16 limbs, whereas the five observed sixth-ranked branches supplied flexor digitorum superficialis. These findings describe this sample; sparse later ranks should not be interpreted as universal anatomical patterns.

Proximodistal rank provides a descriptive way to organize branch origins, but its association with longitudinal position does not independently establish reproducibility. Distinguishing direct origins from downstream divisions prevents duplication of shared coordinates and represents the multi-muscle pathways identified in this study. Recognition of those pathways may inform surgical assessment, but coordinates alone cannot establish muscular selectivity, safe branch sacrifice, or expected functional outcomes. Clinical benefit and measurement reproducibility require separate validation.

## Figures and Tables

**Table 1:** Topography and downstream-branch complexity of muscular branches by proximodistal order.

Order	Branches, *n*	Donors, *n*	RPX, Mean (95% CI)	RPY, Mean (95% CI)	Subbranches, Median (IQR)
1	16	16	0.483 (0.398–0.567)	0.032 (−0.004–0.070)	2 (1–3.5)
2	16	16	0.529 (0.432–0.627)	0.098 (0.079–0.117)	4 (3–6.75)
3	15	15	0.623 (0.518–0.727)	0.226 (0.158–0.307)	4 (2–7.5)
4	12	12	0.737 (0.578–0.914)	0.296 (0.178–0.431)	2 (1–4.5)
5	6	6	0.709 (0.401–0.937)	0.253 (0.167–0.330)	1.5 (1–4.25)
6	5	5	0.876 (0.418–1.156)	0.440 (0.215–0.618)	1 (1–1)

Note: A muscular branch contributes one origin coordinate regardless of the number of downstream records. Confidence intervals were obtained from 10,000 donor-clustered bootstrap resamples.

**Table 2:** Mixed-effects models of normalized muscular-branch origin coordinates.

Coordinate	β per Order	95% CI	*p*	Marginal R^2^	Conditional R^2^	Donor ICC
RPX	0.077	0.050–0.103	<0.001	0.183	0.653	0.576
RPY	0.084	0.063–0.104	—	0.444	0.586	0.255

Note: Models included muscular-branch order as a fixed continuous effect and donor as a random intercept and were estimated by REML. β is the normalized coordinate change associated with a one-order increase. Wald 95% confidence intervals and two-sided Wald *p* values are reported for inferential fixed-effect estimates. Because the RPY association is partly inherent in the definition of rank, its *p* value is not presented and the coefficient is interpreted descriptively. ICC denotes donor-level clustering, not interobserver measurement reliability.

**Table 3:** Number and percentage of muscular branches associated with each target muscle, by branch order.

Order	PT	FCR	PL	FDS	FDP	FPL	PQ	FPL-G
1	16/16 (100.0%)	5/16 (31.2%)	4/16 (25.0%)	4/16 (25.0%)	1/16 (6.2%)	1/16 (6.2%)	1/16 (6.2%)	0/16 (0.0%)
2	7/16 (43.8%)	5/16 (31.2%)	4/16 (25.0%)	12/16 (75.0%)	4/16 (25.0%)	4/16 (25.0%)	3/16 (18.8%)	2/16 (12.5%)
3	1/15 (6.7%)	4/15 (26.7%)	1/15 (6.7%)	9/15 (60.0%)	7/15 (46.7%)	6/15 (40.0%)	6/15 (40.0%)	2/15 (13.3%)
4	0/12 (0.0%)	1/12 (8.3%)	1/12 (8.3%)	7/12 (58.3%)	4/12 (33.3%)	4/12 (33.3%)	4/12 (33.3%)	0/12 (0.0%)
5	0/6 (0.0%)	1/6 (16.7%)	1/6 (16.7%)	6/6 (100.0%)	1/6 (16.7%)	1/6 (16.7%)	1/6 (16.7%)	0/6 (0.0%)
6	0/5 (0.0%)	0/5 (0.0%)	0/5 (0.0%)	5/5 (100.0%)	0/5 (0.0%)	0/5 (0.0%)	0/5 (0.0%)	0/5 (0.0%)

Note: Values are *n*/N (%), where N is the number of muscular branches observed at each order. One muscular branch may contribute to several target columns. PT, pronator teres; FCR, flexor carpi radialis; PL, palmaris longus; FDS, flexor digitorum superficialis; FDP, flexor digitorum profundus; FPL, flexor pollicis longus; PQ, pronator quadratus; FPL-G, Gantzer fascicle.

## Data Availability

The data presented in this study are available from the corresponding author upon reasonable request. The data are not publicly available because they form part of an ongoing line of anatomical research.

## Supplementary Materials

The following supporting information can be downloaded at: https://www.mdpi.com/article/10.3390/brainsci16101068/s1. Video S1: Representative dissection video showing the muscular branches arising from the median nerve and their downstream ramifications toward the target muscles. https://drive.google.com/file/d/1dd9Q_ACefKccIhxiyjUK5_LsnQ_rF1BB/view?usp=sharing (accessed on 1 October 2026).

## Abbreviations

The following abbreviations are used in this manuscript: AIN, anterior interosseous nerve; CI, confidence interval; FCR, flexor carpi radialis; FDP, flexor digitorum profundus; FDS, flexor digitorum superficialis; FPL, flexor pollicis longus; FPL-G, Gantzer fascicle of the flexor pollicis longus; ICC, intraclass correlation coefficient; IQR, interquartile range; PL, palmaris longus; PQ, pronator quadratus; PT, pronator teres; RPX, normalized transverse coordinate; RPY, normalized longitudinal coordinate; R^2^, coefficient of determination.

## References

[1] Caetano E.B., Vieira L.A., Cavalheiro C.S., Arcuri M.H., Sabongi R.G. (2019). Anatomical study of the transfer of the flexor digitorum superficialis nerve branch of the median nerve to restore wrist extension and forearm pronation. Einstein.

[2] Canovas F., Mouilleron P., Bonnel F. (1998). Biometry of the muscular branches of the median nerve to the forearm. Clin. Anat..

[3] Caetano E.B., Toledo J.P.N., Amaral Júnior S.A., Vieira L.A., Pigossi B.D., Andrade R.A. (2020). Innervation of the median nerve motor branches in the forearm and its clinical significance. Acta Ortop. Bras..

[4] Parot C., Leclercq C. (2016). Anatomical study of the motor branches of the median nerve to the forearm and guidelines for selective neurectomy. Surg. Radiol. Anat..

[5] Liu J., Pho R.W.H., Pereira B.P., Lau H.K., Kumar V.P. (1997). Distribution of primary motor nerve branches and terminal nerve entry points to the forearm muscles. Anat. Rec..

[6] El-Din Safwat M.D., Abdel-Meguid E.M. (2007). Distribution of terminal nerve entry points to the flexor and extensor groups of forearm muscles: An anatomical study. Folia Morphol..

[7] Roberts C., Crystal R., Eastwood D.M. (2006). Optimal injection points for the neuromuscular blockade of forearm flexor muscles: A cadaveric study. J. Pediatr. Orthop. B.

[8] Ye J.F., Lee J.H., An X.C., Lin C.H., Yue B., Han S.-H. (2011). Anatomic localization of motor entry points and accurate regions for botulinum toxin injection in the flexor digitorum superficialis. Surg. Radiol. Anat..

[9] Park J., Sohn D., Ko Y.J. (2023). Anatomical location of motor branch points in the flexor digitorum superficialis for the injection site of botulinum toxin. Surg. Radiol. Anat..

[10] Hwang K., Jin S., Hwang S.-H., Lee K.-M., Han S.-H. (2007). Location of nerve entry points of flexor digitorum profundus. Surg. Radiol. Anat..

[11] Jeon A., Lee M., Kim D.W., Kwon O.-Y., Lee J.-H. (2022). Anatomical study of the anterior interosseous nerve. Folia Morphol..

[12] Asghar A., Jha R.K., Patra A., Chaudhary B., Singh B. (2022). The prevalence and distribution of the variants of Gantzer’s muscle: A meta-analysis of cadaveric studies. Anat. Cell Biol..

